# DNA Copy Number Changes in Diffuse Large B Cell Lymphomas

**DOI:** 10.3389/fonc.2020.584095

**Published:** 2020-12-02

**Authors:** Luciano Cascione, Luca Aresu, Michael Baudis, Francesco Bertoni

**Affiliations:** ^1^Institute of Oncology Research, Faculty of Biomedical Sciences, USI, Bellinzona, Switzerland; ^2^SIB Swiss Institute of Bioinformatics, Lausanne, Switzerland; ^3^Department of Veterinary Science, University of Turin, Grugliasco, Italy; ^4^Department of Molecular Life Science, University of Zurich, Zurich, Switzerland; ^5^Oncology Institute of Southern Switzerland, Bellinzona, Switzerland

**Keywords:** copy number aberrations, genetic alteration, lymphoma, diffuse large B cell lymphoma, hematological malignancies, MYC, TP53, CDKN2A

## Abstract

Copy number aberrations (CNV/CNA) represent a major contribution to the somatic mutation landscapes in cancers, and their identification can lead to the discovery of oncogenetic targets as well as improved disease (sub-) classification. Diffuse large B cell lymphoma (DLBCL) is the most common lymphoma in Western Countries and up to 40% of the affected individuals still succumb to the disease. DLBCL is an heterogenous group of disorders, and we call DLBCL today is not necessarily the same disease of a few years ago. This review focuses on types and frequencies of regional DNA CNVs in DLBCL, not otherwise specified, and in two particular conditions, the transformation from indolent lymphomas and the DLBCL in individuals with immunodeficiency.

## Introduction

Copy number aberrations (CNV/CNA) represent a major contribution to the somatic mutation landscapes in cancers, and their identification can lead to the discovery of oncogenetic targets as well as improved disease (sub-) classification (1, 2). In malignant lymphomas, the contribution of partial and complete chromosomal CNV had been recognized early on through cytogenetic analyses (3, 4) and interphase fluorescence in-situ hybridization (FISH) studies (5, 6). The more systematic, genome-wide mapping of CNVs has been facilitated through the development of chromosomal comparative genomic hybridization (CGH) (7, 8) followed by array-based CGH technologies (aCGH) (9, 10) with increasingly higher spatial resolution, as well as through the widespread adoption of SNP-arrays (11) for copy number profiling. More recently the application of high throughput sequencing approaches (12, 13) has led to increasingly precise identification of regional gains or losses of genomic material (14–21), although the frequently used whole-exome sequencing strategies (WES) have limited precision for CNV mapping (22) compared to high-resolution genomic array technologies or whole-genome sequencing WGS). Diffuse large B cell lymphoma (DLBCL) is the most common lymphoma in Western Countries and up to 40% of the affected individuals still succumb to the disease (23–26). DLBCL is an heterogenous group of disorders as it has been demonstrated by studies that have explored transcriptome profiles and/or at DNA alterations in large series of cases (2, 3, 5, 7, 12, 17, 19, 23, 27–40). It is important to mention that the disease we call DLBCL is not necessarily the same of what we called DLBCL just a few years ago. Indeed, the so called “double” or “triple hit lymphomas”, a subgroup of cases with particularly poor prognosis and previously largely included within DLBCL, are now regarded a distinct entity (“High-grade B-cell lymphoma with MYC and BCL2 and/or BCL6 rearrangements”) separate from the “DLBCL, not otherwise specified (NOS)” as expressed in the 2017 WHO classification (24, 31, 39, 41–43). A similar path was previously followed for primary mediastinal B-cell lymphoma (PMBCL), which, based on its very peculiar features (44, 45), was separated from DLBCL and it is considered a distinct clinicopathologic entity (24). Here, we will review the DLBCL genomics with a particular focus on types and frequencies of regional DNA CNVs in DLBCL, not otherwise specified and in two particular conditions, the transformation from indolent lymphomas and the DLBCL in individuals with immunodeficiency.

## CNVs and DLBCL

**Figure 1:**
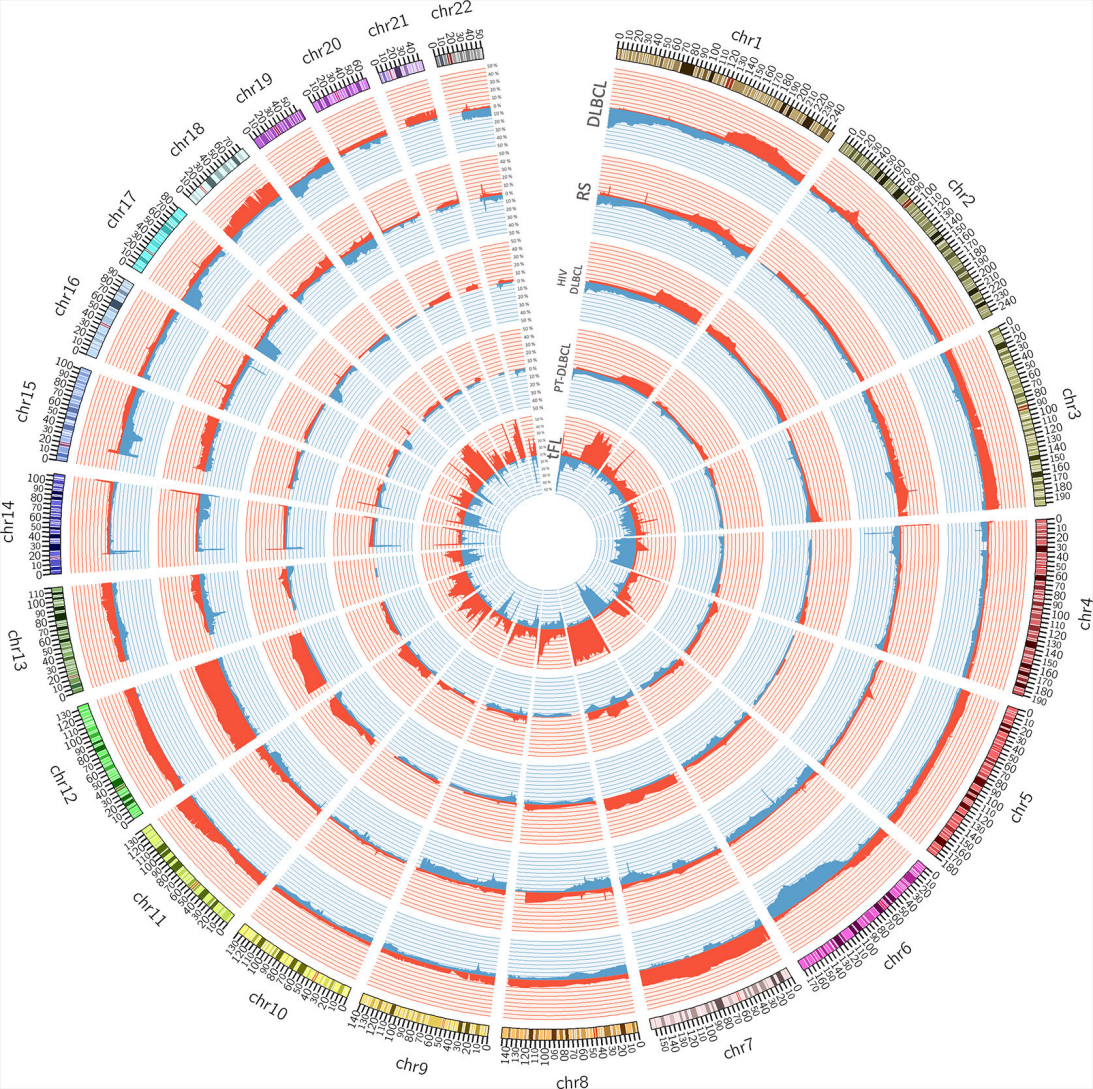
Circos plot summarizing all the copy number changes observed in *de novo* DLBCL (n. = 22), Richter syndrome (RS, n. = 59), HIV-DLBCL (n. = 50), PT-DLBCL (n. = 44), and transformed FL (tFL, n. = 79). For each histology, the layers represent the frequency of copy number loss (blue) and gain (red). Data are obtained from published papers (28, 30, 33, 57, 58). The plot has been generated using Circos tool (v. 0.69) (53).

Within DLBCL, at least two main subtypes have been recognized, in which the gene expression profiles show similarities with two types of normal B-cells: the germinal center B-cell like (GCB) subtype and an activated B cell-like (ABC) subtype (15, 46–51). Clinically, those subtypes are characterized by prognostic differences; patients with an ABC DLBCL have a worse outcome than those with GCB DLBCL when treated with the standard chemo-immunotherapy chemotherapy regimen R-CHOP (24, 48, 49). Genetically, GCB and ABC DLBCL present a series of subtype-specific lesions that explain can explain the different biology of the disease, but they also share others that, with a couple of exceptions (*BCL6* and *MEF2B* alterations), are not DLBCL specific and can be observed in other lymphoma types or even in other cancers. Both GCB and ABC DLBCL present genetic alterations on genes encoding chromatin modifiers [*KMT2D*/MLL2 or *KMT2C*/MLL3 (mutations); *CREEBBP* (mutations or 16p13 deletions) or *EP300* (mutations or 22q13 deletions)], the germinal center master regulator BCL6 (*BCL6* chromosomal translocations, *MEF2B* mutations), proteins involved in DNA damage response ([*TP53* (mutations or 17p13 deletions)], or proteins contributing to immune surveillance [*B2M* (mutations or 15q21 deletions); *CD58* (mutations or 15q21 deletions)]. ABC DLBCL is characterized by lesions in genes involved in NF-κB pathway and B-cell receptor (BCR) signaling [*TNFAIP3* (mutations or 6q23 deletions); *MYD88*, *CD79A*, *CD79B*, *CARD11* (mutations)], cell cycle [*CDKN2A/B* (9p21 deletions)], terminal B cell differentiation [*PRDM1* (mutations or 6q21 deletions); *SPIB* (19q13 gains and amplifications)], and apoptosis [*BCL2* (18q21 gains or amplifications)]. In addition, ABC DLBCL have common gains affecting chromosome 3, which could might contribute to immune escape (*FOXP1*, 3p14), NF-κB pathway activation (*NFKBIZ*, 3q12) and B cell differentiation arrest (*BCL6*, 3q27) (4, 7, 13, 15, 17, 27, 30–32, 34, 36, 38, 40, 48, 49, 52–54). GCB DLBCL presents lesions leading to deregulated cell motility [*GNA13* (mutations)], apoptosis [*BCL2* (chromosomal translocations)], cell cycle [*MYC* (chromosomal translocations)], chromatin regulation [*EZH2* (mutations)], immune escape *TNFRSF14* (mutations or 1p36 deletions), PI3K/AKT signaling [*PTEN* (10q23 deletions); *MIR17HG* (13q31 gains or amplifications)], and DNA damage response [*ING1* (deletions)]. As for ABC DLBCL, also GCB DLBCL present some recurrent gains affecting specific (gains of 2p16 with *REL*) or large and still not fully characterized regions (chromosomes 7 and 12) (15, 16, 49–51, 55, 56). **Figure 1** shows examples of genomic profiles obtained in DLBCL.

The inferior outcome given by the ABC COO alongside the discovery of pathways specifically deregulated in this subtype led to clinical studies designed to target the activation of NF-κB pathway activation. Unfortunately, no advantages for the experimental arms were observed in any of the phase III trials that were looking for improvements in patients classified as ABC DLBCL using gene-expression profiling (34, 59, 60). A possible explanation of these negative results could be not only that treatments that have been explored are not optimal but also that the GCB and ABC subtypes defined at RNA level still comprise too heterogenous patients populations. The latter possibility is strongly sustained by recent studies that have looked at the genetic heterogeneity of DLBCL patients and have led to three novel subclassifications (19–21, 54).

A first classification identifies five clusters (C1-C5) (19) (**Table 1**). **C1** (18% of DLBCL) has cases with BCL6 chromosomal translocations, active NOTCH signaling (*NOTCH2* mutations, *SPEN* inactivation), active NF-κB pathway (*TNFAIP3* mutations or deletions, *BCL10* mutations), and immune escape mechanisms (inactivation of *CD70*, *CD58*, *FAS*, and structural variations of PD-L1 and PD-L2). **C2** (21% of DLBCL) is a mixture of GCB and ABC DLBCL, which share lesions in genes involved in the DNA damage response (*TP53* inactivation), cell cycle (inactivation of *CDKN2A* and *RB1*), PI3K/AKT signaling (*MIR17HG* amplifications), and apoptosis (*MCL1* gain or amplifications). **C3** (13% of all DLBCL) includes GCB-DLBCL with lesions affecting chromatin regulation (*EZH2* mutations, *KMT2D* mutations, *CREBBP* or *EP300* mutations or deletions), PI3K/AKT signaling (*PTEN* deletions or mutations, mTOR mutations, *MIR17HG* amplifications), apoptosis (*BCL2* chromosomal translocations), cell motility (*GNA13* mutations), and germinal center program (*MEF2B* or *IRF8* mutations). The GCB DLBCL **C4** (17% of all DLBCL) contains cases with genetic lesions affecting chromatin structure (mutations in linker and core histone genes), immune escape (*CD83*, *CD58*, and *CD70*), NF-κB pathway (mutations of *CARD11*, *NFKBIE*, and *NFKBIA*), BCR and PI3K signaling (mutations of *RHOA* and *SGK1*), cell motility (*GNA13* mutations), and RAS/JAK/STAT signaling (*BRAF* and *STAT3* mutations). The last one, **C5** (21% of all DLBCL) comprises ABC DLBCL cases with *BCL2* gains, concordant *MYD88* L265P/*CD79B* plus additional lesions such as gains of 3q, 19q13.42 and inactivation of *PRDM1*.

**Table 1:** DLBCL subtypes according to Chapuy et al. (19).

DLBCL subtype	COO	%	MUTATIONS	GENOMIC LESIONS
C1	ABC	18%	*BCL10, TNFAIP3, UBE2A, CD70, B2M, NOTCH2, TMEM30A, FAS, ZEB2, HLA-B, SPEN, PDCD1LG2/CD274*	GAINS: +5pFUSIONS AND TRANSLOCATIONS: 3q27 (*BCL6*), 9p24 (*PDCD1LG2*/*CD274*), 3q28 (*TP63*)
C2	ABC/GCB	21%	*TP53*	GAINS: +1q23 (*MCL1*), +13q31 (*MIR17HG*), plus additional gross aberrations.LOSSES: -17p13 (*TP53)*, -9p21 (*CDKN2A*), -13q14 (*RB1*), -1q42, plus additional gross aberrations.
C3	GCB	13%	*BCL2, CREBBP, EZH2, KMT2D, TNFRSF14, HVCN1, IRF8, GNA13, MEF2B, PTEN*	LOSSES: -10q23 (*PTEN*).FUSIONS AND TRANSLOCATIONS: 18q21 (*BCL2*).
C4	GCB	17%	*SGK1, HIST1H1E, NFKBIE, BRAF, CD83, NFKBIA, CD58, HIST1H2BC, STAT3, HIST1H1C, ZFP36L1, KLHL6, HIST1H1D, HIST1H1B, ETS1, TOX, HIST1H2AM, HIST1H2BK, RHOA, ACTB, LTB, SF3B1, CARD11, HIST1H2AC*	–
C5	ABC	21%	*CD79B, MYD88, ETV6, PIM1, TBL1XR1, GRHPR, ZC3H12A, HLA-A PRDM1, BTG1*	GAINS: +18q (*BCL2*, *MALT1*), +3q, +18p, +3p, +19q13.42, +19q.LOSSES : -17q25.1, -19p13.2, -6q21 (*PRDM1*).
C0*	ABC	4%	–	–

*unclassified.

The second classification originally identified four subtypes (EZB, MCD, N1, and BN2) (20), which more recently have been extended to six (21) (**Table 2**). Cluster **EZB** (22% of DLBCL) resembles C3 and the genomic lesions of GCB DLBCL with lesions in genes coding for proteins involved in chromatin regulation (*EZH2* mutations, *KMT2D* mutations, *CREEBBP* or *EP300* mutations or deletions), apoptosis (BCL2 translocations), immune escape (*TNFRSF14* mutations or deletions), cell motility (*GNA13* mutations), JAK/STAT signaling (STAT6 mutations or amplifications, *SOCS1* mutations or deletions), PI3K/AKT signaling (*PTEN* deletions, mTOR mutations, and *MIR17HG* amplifications), immune escape (inactivation of TNFRSF14, *CIITA*, HLA-DMA), and *REL* amplifications. The **MCD** cluster (8% of DLBCL), similar to the C5, contains almost exclusively ABC-DLBCL with aberrant activation of the chronic BCR and NF-κB signaling (mutations of *MYD88*, *CD79A*, *CD79B*, and *CARD11*), impaired terminal B cell differentiation (*PRDM1* mutations or deletions, *SPIB* gains or amplifications), deregulated cell cycle (*CDKN2A/B* deletions), and immune escape (mutations or deletions of HLA-A, HLA-B, HLA-C, and *CD58*). The **N1** subtype (2% of DLBCL) mostly contains ABC DLBCL with Notch activation (*NOTCH1* mutations), NF-κB pathway (TNFAIP3 mutations or deletions), and impaired terminal B cell (lesions of *IRF4*, *ID3*, and *BCOR*). The **BN2** (15% of DLBCL), similar to C1, contains both GCB and ABC DLBCL and it is enriched of cases with Notch activation (*NOTCH2* mutations or amplifications, mutations of *DTX1* or *SPEN*), BCL6 translocations, NF-κB signaling (inactivation of *TNFAIP3* or *TN1P1* and gains or amplification of *PRKCB* and *BCL10*), immune escape (*CD70* inactivation), cell cycle (*CCND3* mutations), and cell migration (*CXCR5*). Since with this classification almost half of DLBCL cases did not fit in any defined subgroup (20), two additional subtypes have been proposed (ST2 and A53) (21). The **ST2** subtype (6% of DLBCL) is consists mostly of GCB DLBCL and is characterized by mutations in *TET2*, *SGK1* and JAK/STAT (*SOCS1* and *STAT3* mutations), and homing effectors (*GNA13* and *P2RY8*). The **A53** subtype is enriched of ABC DLBCL and is characterized by *TP53* mutations and deletions, with extensive aneuploidy, plus deletions of the *B2M* locus, amplifications of *CNPY3* (6p21), 6q losses (*TNFAIP3* and *PRDM1*), gain/amplification of 3q (*NFKBIZ*) and *BCL2* amplifications. Moreover, following the development of a double-hit gene expression signature identifying GCB-DLBCL patients with no evidence of a dual hit at FISH analysis but an outcome similar to the double-hit patients (36), the EZB group has been divided based on the presence (EZB-MYC+) or absence (EZB-MYC-) of a double hit (DHIT) signature (21).

**Table 2:** DLBCL subtypes according to Wright et al. (21).

DLBCL subtype	COO	%	MUTATIONS	GENOMIC LESIONS
MCD	ABC	9%	*MYD88* L265P*, CD79B, PIM1/2, HLA-A/B/C, BTG1/2, CDKN2A, ETV6, OSBPL10, TOX, MPEG1, SETD1B, KLHL14, TBL1XR1, GRHPR, PRDM1, CD58, TAP1, FOXC1, IRF4, VMP1, SLC1A5, DAZAP1, BCL11A, PPP1R9B, IL10RA, IL16, CHST2, ARID5B, WEE1, KLHL42 TNRC18*	GAINS: +18q21 (*BCL2*), +19q13 (*SPIB*, *SLC1A5*),+19p13 (*DAZAP1*).LOSSES: -6p21 (*HLA*-*A/B*/*C*, *TAP1*), -8q12 (TOX), -6q21 (*PRDM1*), - 1p13 (*CD58*), -9p21 (*CDKN2A*).FUSIONS AND TRANSLOCATIONS: 9p24 (*PDCD1LG2*/*CD274*).
EZB	GCB	20%	*EZH2, TNFRSF14, KMT2D, CREBBP, FAS, IRF8, EP300, MEF2B, CIITA, ARID1A, GNA13, STAT6, EBF1, GNAI2, C10orf12, BCL7A, HLA-DMB, S1PR2, MAP2K1, FBXO11*	GAINS: +2p16 (*REL*), chromosome 12p, + 12q13 (*STAT6*), chromosome 21, + 13q31 (*MIR17HG*)LOSSES: -10q23 (*PTEN*), -1p36 (*TNFRSF14*, *ARID1A*), - 12q13 (*KMT2D*), -16p13 (*CREBBP*, CIITA), - 10q24 (*FAS*), -22q13 (*EP300*), -17q24 (*GNA13*), -5q33 (*EBF1*), -10q24 (*C10ORF12*), -15q22 (*MAP2K1*), -2p16 (*FBXO11*).FUSIONS AND TRANSLOCATIONS: 18q21 (*BCL2*), 16p13 (*CIITA*).
N1	ABC	2%	*NOTCH1, IRF2BP2, ID3, BCOR, EPB41, IKBKB, ALDH18A1*	GAINS: 4p.
BN2	ABC/GCB	13%	*NOTCH2, TNFAIP3, DTX1, CD70, BCL10, UBE2A, TMEM30A, KLF2, SPEN, CCND3, NOL9, TP63, ETS1, HIST1H1D, PRKCB, HIST1H2BK, TRIP12, KLHL21, TRRAP, PABPC1*	GAINS: +1p12 (*NOTCH2*), +1p22 (*BCL10*), +16p12 (*PRKCB*).LOSSES: -6q23 (*TNFAIP3*), -6q14 (*TMEM30A*), -1p36 (*SPEN*), -3q28 (TP63).FUSIONS AND TRANSLOCATIONS: 3q27 (*BCL6*).
ST2	GCB	6%	*TET2, SGK1, DUSP2, ZFP36L1, ACTG1, ACTB, ITPKB, NFKBIA, STAT3, EIF4A2, JUNB, BCL2L1, DDX3X, SOCS1, CD83, P2RY8, RFTN1, RAC2, XBP1, SEC24C, MED16, PRRC2C, EDRF1, DOCK8, CLTC, ZNF516, WDR24, ZC3H12D*	LOSSES: -16p13 (*SOCS1*).
A53	ABC/GCB	6%	*TP53, B2M, TP53BP1, TP73*	GAINS: +6p21 (*CNPY3*), +3q12 (*NFKBIZ*), plus additional gross aberrations.LOSSES: -17p13 (*TP53*), -15q21 (*B2M*), -15q15 (*TP53BP1*), -13q34 (*ING1*), 1p36 (*TP73*), plus additional gross aberrations.
unclassified	ABC/GCB	37%	–	–

Starting from a series of 928 cases that included also not *de novo* DLBCL and that were analyzed with a targeted panel of 293 genes, the last classification identifies five subgroups, with names based on their most common lesion (*MYD88*, *BCL2*, *SOCS1/SGK1*, *TET2/SGK1*, and *NOTCH2*), leaving 27% of cases unclassified (54) (**Table 3**). The **MYD88** cluster (16%) contains mostly ABC, and genes commonly mutated are *MYD88* (L265P), *PIM1*, *CD79B*, and *ETV6* with also *CDKN2A* losses. The cluster overlaps with C5 and MCD from the other classifications (19, 21) and contains primary extranodal DLBCL (CNS; testis, breast). The **BCL2** cluster (19%) includes mostly GCB DLBCL and the majority of the cases that bear a *BCL2* translocation. It has high frequency of mutations of *EZH2*, *BCL2*, *CREBBP*, *TNFRSF14*, *KMT2D*, and *MEF2B*. The cluster overlaps with previously described C3 and EZB (19, 21) and contains most of the transformed FL included in the series. The **SOCS1/SGK1** group (12%) presents mutations of *SOCS1*, *CD83*, *SGK1*, *NFKBIA*, *HIST1H1E*, and *STAT3*. The **TET2/SGK1** cluster (11%) includes cases with mutations of *TET2*, *SGK1*, *KLHL6*, *ZFP36L1*, *BRAF*, *MAP2K1*, and *KRAS*. Both the SOCS1/SGK1 and the TET2/SGK1 clusters contain mostly GCB and overlap with the ST2 and C4 of the other classifications. Importantly, the SOCS1/SGK1 cluster also includes the PMBCL cases included in the study (*STAT3* and *SOCS1* mutations). The last cluster (**NOTCH2**, 15%) presents mutations of *NOTCH2*, *BCL10*, *TNFAIP3*, *CCND3*, *SPEN*, *TMEM30A*, *FAS*, and *CD70*, and cases with *BCL6* translocations. It has both GCB and ABC and it overlaps with the previously reported BN2 and C1 clusters (19, 21).

**Table 3:** DLBCL subtypes according to Lacy et al. (54).

DLBCL subtype	COO	%	MUTATIONS	GENOMIC LESIONS*
MYD88	ABC	16%	*MYD88, PIM1, CD79B, ETV6*	LOSSES: -9p21 (*CDKN2A*).
BCL2	GCB	19%	*EZH2, BLC2, CREBBP, TNFRSF14, KMT2D*	FUSIONS AND TRANSLOCATIONS: 18q21 (*BCL2*),
SOCS1/SGK1	GCB	12%	*SOCS1, CD83, SGK1, NFKBIA, HIST1H1E*	–
TET2/SGK1	GCB	11%	*TET2, BRAF, SGK1, KLHL6, ID3*	–
NOTCH2	ABC/GCB	15%	*NOTCH2, BLC10, TNFAIP3, CCND3, SPEN*	FUSIONS AND TRANSLOCATIONS: 3q27 (*BCL6*).
unclassified	ABC/GCB	27%	–	–

*the study performed targeted DNA sequencing on all the cases, while FISH analyses for BCL2 and BCL6 translocations were not done in all the cases done (54). CDKN2A data, based on sequencing data.

Although similar genetic features are picked up by the three classifications (19–21, 54), the final overlaps are only partial (**Table 4**), largely due to the approaches used by the Investigators to tackle the issue of DLBCL heterogeneity. However, the two large ABC and GCB subtypes have now been split in subgroups of cases bearing more similar genomic landscapes, and, thus, perhaps sharing more similar responses to targeted therapies (**Table 4**). New generation of clinical trials can now be designed to assess targeted agents, for example in addition to R-CHOP, in much better genetically defined subgroups of patients.

**Table 4:** Overlaps among DLBCL classifications and potential therapeutic interventions.

Cell of origin (46, 47)	DFCI/HMS * (19)	NCI** (20, 21),	HMRN*** (54)	Potential therapeutic interventions^
ABC	C5	MCD	MYD88 *	Lenalidomide; BTK inhibition; IRAK4 inhibition; BET inhibition; PI3K/mTOR inhibition; JAK/STAT inhibition; PKCβ inhibition BCL2 and BCL-XL inhibition
ABC	*	N1	*	Immune checkpoints; Notch1 inhibition
GCB	C3	EZB	BCL2	PI3K/mTOR inhibition; EZH2 inhibition; BCL2 inhibition; MYC inhibition; CREBBP inhibition
GCB	C4	*	SOCS1/SGK1	JAK/STAT inhibition; BRAF/MEK1 inhibition
GCB	*	ST2	TET2/SGK1	PI3K inhibition; JAK2 inhibition
GCB/ABC	C1	BN2	NOTCH2	BET inhibition; PI3K/mTOR inhibition; Lenalidomide; NF-kB inhibition; PKCβ inhibition; BCL2 inhibition; Notch2 inhibition
GCB/ABC	C2	A53	NEC	NF-kB inhibition; CDK inhibition

*Dana-Farber Cancer Institute/Harvard Medical School; **National Cancer Institute; ***Haematological Malignancy Research Network; ^(19–21).

Interestingly, the genetics of the individual subtypes suggest that some DLBCL derive from the transformation of indolent lymphomas and/or that they follow specific pathogenetic mechanisms at least partially shared by other lymphoid neoplasms. These connections are evident for C5, MCD, MYD88 (primary extranodal DLBCL of the central nervous system or of the testis; transformed Waldenström macroglobulinemia), C3, EZB, BCL2 [follicular lymphoma (FL); transformed FL; Burkitt lymphoma), N1 [(NOTCH1 mutated chronic lymphocytic leukemia (CLL)], C2, BN2, NOTCH2 (transformed MZL), and ST2 (nodular lymphocyte-predominant Hodgkin lymphoma; T cell/histiocyte-rich large B cell lymphoma) (19–21, 54).

## Copy Number Changes and Transformation From Indolent Lymphomas to DLBCL

Copy number changes play important role in the transformation from indolent lymphomas to DLBCL and their presence can also be associated with a higher risk of transformation. Deregulation of MYC *via* DNA gains, amplifications or chromosomal translocation is the most frequent event occurring at the transformation from FL to DLBCL, followed by inactivation, mainly by DNA loss, of *CDKN2A/B*, of *B2M* (losses or mutations) and activating mutations of *P1M1* (28, 61). Transformed FL also have higher frequency of 3q and 11q gains than FL (28). Transformed FL and GCB DLBCL are phenotypically similar but their genomic profiles are not the same (28). Here, they present similar frequencies of 1p losses and 2p gains, but overall fewer occurrences of 13q gains (*MIR17HG*) or losses (*ING1*), as well of *PTEN* losses at 10q. Deletions of *TNFAIP3* and of *CDKN2A* are more common in transformed FCL than in GCB DLBCL (28).

**Figure 2:**
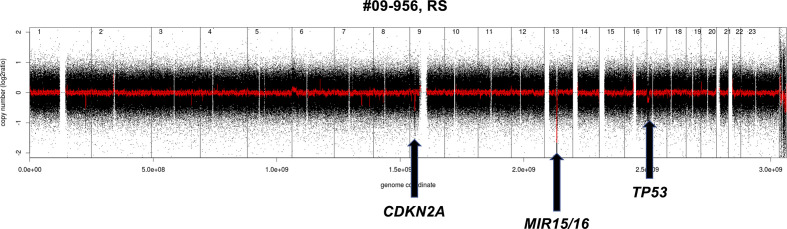
Example of genomic profile of a RS case bearing the typical *CDKN2A* and *MIR15/16* deletion. Profiles obtained using the Affymetrix Genome-Wide Human SNP Array Version 6.0 [modified from (33)]. Black, raw copy number values; red, smoothed copy number values. X-axis, genomic mapping; Y-axis, log2 copy-number values.

A quite similar pattern is observed in the transformation from CLL to DLBCL (Richter syndrome) with the deletion at the *CDKN2A/B* locus as the most common acquired event (33, 37, 62). Despite the morphological appearance, as a whole, Richter syndrome has a CNV pattern that differs from *de novo* DLBCL, largely due to the under-representation of DNA gains and losses that are common in the latter disorder. Richter syndrome samples have a higher frequency of deletions at 7q31-q36 (still undefined role) and of the CLL related losses at 13q14.3 and 11q22.3 as well as trisomy 12 (**Figure 2**). Interestingly, copy number changes define two main subtypes of Richter syndrome (33). A first group (50% of Richter syndrome) bears *TP53* inactivation (by loss or by somatic mutations) and/or *CDKN2A* loss, alongside *MYC* gain/amplifications, 13q14.3 loss and additional lesions (33). A second group has almost exclusively trisomy 12 (33).

Regarding the risk of transformation to DLBCL, deletions at 1p35, 6q and copy neutral LOH at 16p have been associated with higher risk of transformation to DLBCL in FL patients (32, 63). The presence of losses at 17p (*TP53*), 15q (*MGA*), and gains at 2p (*MYCN*, *REL*) and the lack of 13q14.3 deletions targeting *MIR15/MIR16* appeared linked with a higher risk of transformation to Richter syndrome from CLL (33).

## Copy Number Changes and Immunodeficiency-Related DLBCL

As there are differences in recurring CNV patterns between GCB and ABC DLBCL as well as between Richter syndrome and *de novo* DLBCL, a similar observation can be made when comparing the genomic profiles of DLBCL in immunocompetent individuals versus immunodeficiency related DLBCL. This became evident from studies comparing DLBCL obtained in persons with human immunodeficiency virus (HIV) infection in the pre-HAART (highly active antiretroviral therapy) (HIV-DLBCL) era, and in recipients of solid organ transplants (PT-DLBCL) with DLBCL from immunocompetent individuals, all analyzed with the same platform and data mining workflow (30, 57). First, a higher frequency of DNA breakages within fragile sites is seen in immunodeficiency related DLBCL than in immunocompetent cases, with perhaps a higher contribution of these changes to the etiology of the disease. Since viral DNA can insert in fragile sites, the immunodeficiency can expose the individuals to a multitude of viruses, which could infect B cells and integrate in the genome, preferentially at fragile sites (35, 38, 40, 52, 64–67).

Despite their phenotypic reminiscence of post-GC B-cells (29, 68), PT-DLBCL have a pattern of DNA gains and losses that is different from ABC DLBCL, lacking gains of 3q and 18q (*BCL2*, *NFATC1*) and losses of 6q (*PRDM1* and *TNFAIP3*) (57). Pre-HAART HIV-DLBCL show genomic profiles that are intermediate between ABC and GCB DLBCL, with more similarities towards the latter. Indeed, HIV-DLBCL has GCB DLBCL lesions such as gains of 2p, 7q, and 12q, as well as losses of 1p, but it also carries 3q and 18q gains, commonly associated with ABC DLBC, and lacks the 10q deletions involving *PTEN* (30).

While gains of 1q, 11q and of chromosome 7 as well as 17p losses are present in both immunodeficiency related and immunocompetent DLBCL, deletions at 13q14 are usually absent (30, 57) suggesting a possible role in immune escape for the inactivation of *MIR15/MIR16* or of *RB1*, whose loci on 13q are frequently co-deleted in DLBCL (69). Interestingly, the loss of RB1 has been associated with T-cells exclusion in prostate cancer (70). Similarly, PT-DLBCL do not show copy neutral LOH (CN-LOH) affecting 6p, a common feature in different lymphomas including DLBCL and HIV-DLBCL. CN-LOH on 6p is believed to contribute to the silencing of the major histocompatibility complex (71) and DLBCL can indeed show absence or reduced expression of MHC class II proteins (27, 71–73). Importantly, the low MHC-II expression is associated with a decreased number of infiltrating T cells and reduced cytotoxic CD8+ T cells activation (27). Thus, it seems that the immune escape induced by the 6p copy neutral LOH is not required by PT-DLBCL but still needed by HIV-DLBCL. This could be due to the iatrogenic immunosuppression lowering both CD4+ and CD8+ T cells in the first lymphoma type while the viral infection causes a more pronounced loss of CD4+ than of cytotoxic CD8+ T-cells. Similarly, PT-DLBCL also have fewer B2M mutations—another immune escape mechanism—than immunocompetent DLBCL (74). It is also worth mentioning that among immunodeficiency related DLBCL the presence of Epstein Barr virus (EBV) is associated with a lower number of genomic lesions, both in terms of copy number changes and of somatic mutations (30, 57, 74, 75).

**Figure 3:**
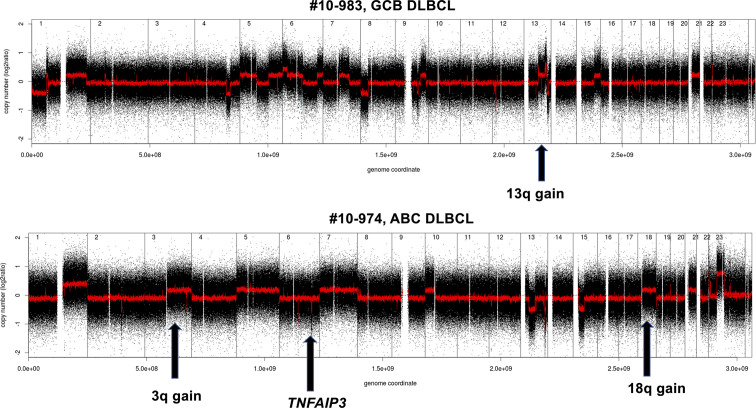
Examples of genomic profile of two DLBCL cases bearing GCB (above) or ABC (below) lesions among others. Profiles obtained using the Affymetrix Genome-Wide Human SNP Array Version 6.0 [modified from (33)]. Black, raw copy number values; red, smoothed copy number values. X-axis, genomic mapping; Y-axis, log2 copy-number values.

A global view of the different genomic profiles of DLBCL, Richter syndrome, immunodeficiency related DLBCL and transformed FL can be seen in **Figure 3**.

## Conclusions

Data obtained in all these last years using genome wide technologies that allow for the molecular study of transcriptome profiles and of DNA changes (CNVs or somatic mutations) have led to building a much more precise framework to explain the heterogenous biology and clinical course of DLBCL cases. Although novel approaches such as the use of liquid biopsies are becoming increasingly feasible at least in the context of clinical trials, reproducible and commonly agreed genetic classification systems have to be defined. This is necessary to compare results from future individual clinical trials and to then transfer the findings to the right patients in the clinical practice. Indeed, the identification of group of patients with homogenous patterns of genetic lesions leading to the deregulation of specific pathways represents an opportunity to study novel agents in a more targeted approach than done so far, hopefully overcoming the disappointing results obtained trying to target the ABC DLBCL subtype defined based on gene expression profiling.

## Author Contributions

All authors participated to the design of the review, literature revision, manuscript writing, and final revision. All authors contributed to the article and approved the submitted version.

## Funding

Partially supported Oncosuisse (02296-08-2008, 1939-8-2006), and Swiss National Science Foundation (Sinergia 147620), Bern, Switzerland.

## Conflict of Interest

The authors declare that the research was conducted in the absence of any commercial or financial relationships that could be construed as a potential conflict of interest.
